# Supplementary material for: Content-rich biological network constructed by mining PubMed abstracts
Source: BMC Bioinformatics. 2004 Oct 8;5:147. doi: 10.1186/1471-2105-5-147 (PMC528731; doi:10.1186/1471-2105-5-147)
Supplement: Additional File 2 — The original results of the above study (non-essential files are deleted to keep the file size under the limit set by BMC bioinformatics). [file 1471-2105-5-147-S2.bz2 › chilibotAdditionalFile2/dip05/154ID7630397E551/html/CDK2_CCNA2.html]

 


 **CDK2** and **CCNA2** 
  
Found 650 abstracts in PubMed, retrieved 05.  
 

 What does Google say? 
 PDF only 
| .edu only 

---

**Interactive relationship** (e.g. stimulation, inhibition, etc)

**Inhibitory relationship**- The inhibition of cyclin A  [ **CCNA2** ]  gene expression is associated with a block in cyclin E  **cdk2**  activity and phosphorylation of both the retinoblastoma protein and p107.  Ref: 12869629 Mol Pharmacol, 2003
**Neutral relationship**

**Non-interactive relationship** (e.g. studied together, co-existance, homology, etc.)

- Cyclin A  [ **CCNA2** ]   **cdk2**  appears to associate with NF Y both in vitro and in vivo.  Ref: 12857729 J Biol ChemJ Biol Chem, 2003
- The roles of cyclin A  [ **CCNA2** ]  E  **CDK2**  complexes in driving replication, their relationship with other regulators of S phase, and their role in keeping replication to only once per cell cycle will be discussed.  Ref: 12851482 Cell Cycle< MedlineTA>Cell Cycle, 2003
- Arsenic increased expression of the P21 protein and decreased levels of cyclin A  [ **CCNA2** ] , cyclin B1 and cyclin D1, but expression of  **CDK2** , CDK4, CDK6, and cyclin E were not affected.  Ref: 12883267 Pancreas, 2003
- Cyclin E  **CDK2**  and cyclin A  [ **CCNA2** ]   **CDK2** , together with DBF4 CDC7, phosphorylate several components of the prereplication complex and replication machinery.  Ref: 12851482 Cell Cycle< MedlineTA>Cell Cycle, 2003
- Furthermore, YA protein is phosphorylated in parallel with a cell cycle dependent activation of  **cdk2**  kinase and cyclin A  [ **CCNA2** ]  expression.  Ref: 12857729 J Biol ChemJ Biol Chem, 2003
- it delayed the activity of the cyclin A  [ **CCNA2** ]   **cdk2**  complex to 34h.  Ref: 12876382 Cell Physiol Biochem, 2003
